# Automatic analysis of selected choroidal diseases in OCT images of the eye fundus

**DOI:** 10.1186/1475-925X-12-117

**Published:** 2013-11-14

**Authors:** Robert Koprowski, Slawomir Teper, Zygmunt Wróbel, Edward Wylegala

**Affiliations:** 1 Department of Biomedical Computer Systems, University of Silesia, Faculty of Computer Science and Materials Science, Institute of Computer Science, ul. Będzińska 39, Sosnowiec 41-200, Poland; 2 Clinical Department of Ophthalmology, District Railway Hospital in Katowice, School of Medicine with the Division of Dentistry in Zabrze, Medical University of Silesia, Katowice, Poland

**Keywords:** Eye, Image processing, OCT, Texture analysis, Conditional erosion and dilation

## Abstract

**Introduction:**

This paper describes a method for automatic analysis of the choroid in OCT images of the eye fundus in ophthalmology. The problem of vascular lesions occurs e.g. in a large population of patients having diabetes or macular degeneration. Their correct diagnosis and quantitative assessment of the treatment progress are a critical part of the eye fundus diagnosis.

**Material and method:**

The study analysed about 1’000 OCT images acquired using SOCT Copernicus (Optopol Tech. SA, Zawiercie, Poland). The proposed algorithm for image analysis enabled to analyse the texture of the choroid portion located beneath the RPE (Retinal Pigment Epithelium) layer. The analysis was performed using the profiled algorithm based on morphological analysis and texture analysis and a classifier in the form of decision trees.

**Results:**

The location of the centres of gravity of individual objects present in the image beneath the RPE layer proved to be important in the evaluation of different types of images. In addition, the value of the standard deviation and the number of objects in a scene were equally important. These features enabled classification of three different forms of the choroid that were related to retinal pathology: diabetic edema (the classification gave accuracy *ACC*_*1*_ = 0.73), ischemia of the inner retinal layers (*ACC*_2_ = 0.83) and scarring fibro vascular tissue (*ACC*_3_ = 0.69). For the cut decision tree the results were as follows: *ACC*_*1*_ = 0.76, *ACC*_*2*_ = 0.81, *ACC*_*3*_ = 0.68.

**Conclusions:**

The created decision tree enabled to obtain satisfactory results of the classification of three types of choroidal imaging. In addition, it was shown that for the assumed characteristics and the developed classifier, the location of B-scan does not significantly affect the results. The image analysis method for texture analysis presented in the paper confirmed its usefulness in choroid imaging. Currently the application is further studied in the Clinical Department of Ophthalmology in the District Railway Hospital in Katowice, Medical University of Silesia, Poland.

## Introduction

Choroid plays an essential role in many physico-chemical processes. The structure is important also for ciliary-retinal vessels (observed in minority of patients) originating from the choroid which supply the speckle field and protect against loss of central vision, for example in the case of central retinal artery (CRA) occlusion [1]. Visible choroidal vessels are found to a lesser extent in foveal avascular zone (FAZ). In the case of fluorescein angiography, it shows no presence of the fluorescence in that area (due to high amount of pigment). Conversely, any leakage of the pigment or FAZ staining indicates macular disease. Fluorescein diagnostic method is, in this case, profiled to carry out this type of diagnosis [2]. In practice, therefore, diagnosis of the eye fundus and choroidal layer using optical coherence tomography (OCT) also brings correct results [3,4]. The analysis of the vascular layer located beneath the RPE layer (retinal pigment ephitelium) in the OCT image of the eye is presented in a small number of publications [4-14]. They are mainly related to qualitative analysis of the choroidal layer without quantitative treatment of the characteristic distributions which occur there. Therefore, in this paper quantitative analysis of the choroidal layer using the new developed algorithm for image analysis and processing was proposed. Most of currently used OCT devices are not intended for choroidal imaging. The authors tried to obtain data resulting from the choroid reflectivity in a wide variety of patients. The algorithm was profiled to the analysis of the following types of images:

–neovascular AMD or exudations secondary to diabetic or thrombotic edema (specific layouts of shadows in the choroid caused by retinal changes) [5,6],

–diffuse macular edema without blood and exudations or ischemia of the inner retinal layers (global reduction of brightness in an OCT image) in such patients there is need to differentiate between the choroidal atrophy due to degeneration or high myopia [7],

–scarring fibrovascular tissue a uniform image proves its presence in most patients [7-9].

These types of images are presented in Table 1 they are subject to further analysis.

**Table 1:** Types of images and their features visible in OCT images

**Symbol**	**Imaging type**	**Features visible in the image**
*Z*_*1*_	neovascular AMD or exudations secondary to diabetic or thrombotic edema	characteristic layouts of shadows in the choroid caused by retinal changes
*Z*_*2*_	diffuse macular edema without blood and exudations or ischemia of the inner retinal layers	global reduction in brightness in the OCT image
*Z*_*3*_	scarring fibrovascular tissue	uniform image is evidence of its presence

## Material

The study analysed about 1’000 OCT images acquired using SOCT Copernicus (Optopol Tech. SA, Zawiercie, Poland). The patients ranged in age from 12 to 78 years and had different types of choroidal structure. It was a group of patients routinely examined, analyzed retrospectively and anonymously. The routine tests were carried out in accordance with the Declaration of Helsinki. The images were acquired in DICOM or RAW format with a resolution of 256×1024 pixels at 8 bits per pixel. Image analysis was carried out in Matlab with Image Acquisition Toolbox and Signal Processing tools (version 4.0 and 7.1 respectively), whereas code optimization was carried out in the C language. The proposed algorithm for image analysis enabled to analyse the texture of the choroid portion located beneath the RPE (Retinal Pigment Epithelium) layer. The analysis of the choroid was performed using the new profiled algorithm based on texture analysis and mathematical morphology that is described below. The division into types of images (3 groups) was carried out by an ophthalmologist for 1′000 images representing learning, validation and test groups (proportion: 60%, 20%, 20% respectively). At this stage, patients with other type of images were also eliminated from further analysis - only images with visible alterations in the choroid were further analyzed.

## Method

### Preprocessing

**Figure 1:**
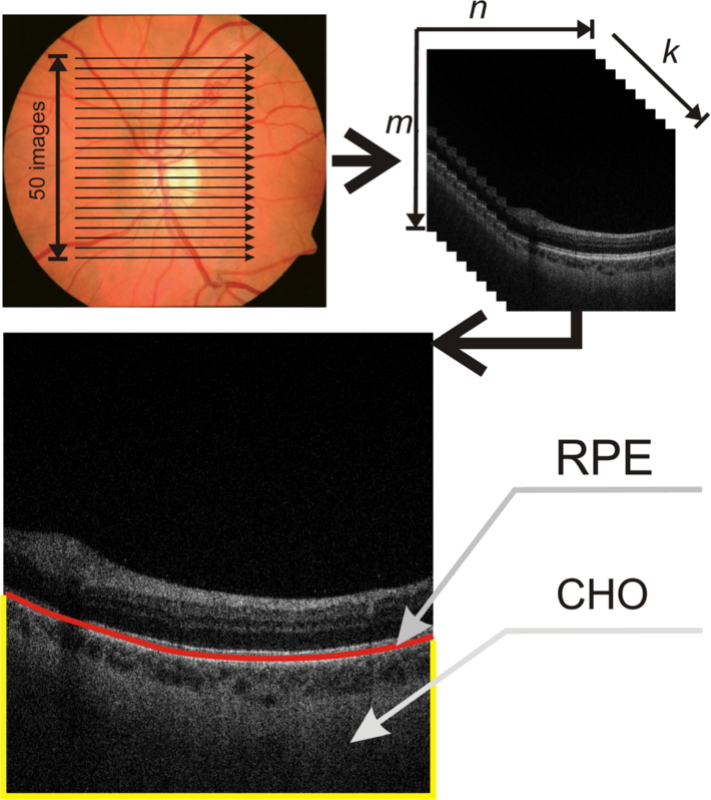
**The method for obtaining tomographic images of the fundus.** For a sample 2D tomographic image, the RPE layer (retina pigment epithelium) and the choroid layer CHO are highlighted. Image analysis applies to the proposed algorithm which analyses the choroid layer using new methods of texture analysis and mathematical morphology. In each case, a flat two-dimensional input image is analysed, whose resolution (and that of the OCT apparatus) does not affect the obtained results.

**Figure 2:**
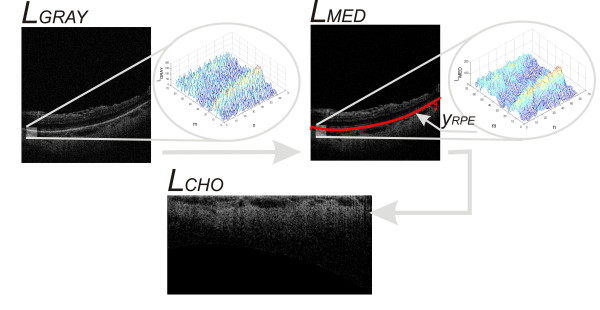
**The course of image pre-processing.** The subsequent steps of *L*_*GRAY*_ image analysis: filtration with a median filter – *L*_*MED*_, determination of the RPE layer (retinal pigment epithelium) – *y*_*RPE*_(*n*), coordinate system conversion – *L*_*CHO*_. These steps are part of the image pre-processing which is necessary for further analysis of images.

The input image *L*_*GRAY*_ with a resolution *M*×*N* = 256×1024 pixels (*M* – number of rows, *N* – number of columns of the image) was subjected to median filtering using a mask *h* sized *M*_*h*_×*N*_*h*_ = 3×3 pixels, thus obtaining the image *L*_*MED*_. The size of the filter mask *h* was chosen on the basis of medical evidence on the extent of artefacts found in this layer of the eye fundus OCT image and resolution of the image *L*_*GRAY*_. The images of successive stages of image pre-processing are shown in Figures 1 and 2. The image *L*_*MED*_ is further analysed to detect the RPE layer *y*_*RPE*_ ' (*n*) [3,15-17]. For this purpose, every *n*-th column of the image *L*_*MED*_ is examined. The position of maximum brightness for each column is determined, i.e.:

(1) yRPE'n=argmaxm∈1,MLMEDm,n

where:

*m,n* – coordinates of rows and columns of the matrix *m*∈(1,*M*) and *n*∈(1,*N*).

The Equation (1) can be directly applied only if for all the analysed rows and successive columns, there is only one maximum value of brightness. In practice, it occurs in about 80% of the analysed cases at the resolution of 8 bits per pixel. The Equation (1) is also very sensitive to noise, especially in the case of single bright pixels – salt and pepper noise. This type of noise is sometimes not fully filtered during median filtering. For this reason, the Equation (1) was expanded to the following form (*y*_*RPE*_ '' (*n*)):

(2) $$L_{\mathrm{MAX}}\left(m,n\right)=\left\{\begin{array}{ccc} m & \mathrm{if} & L_{\mathrm{MED}}\left(m,n\right)<\max_{m\in \left(1,M\right)}L_{\mathrm{MED}}\left(m,n\right)*p_{r}\\ 0 & \mathrm{others} & \end{array}\right)\;$$

(3) yRPE''n=medm∈1,Mm≠0LMAXm,nif∑m=1MLMAXm,nMother>0

where:

*med* - median

*p*_*r*_ - coefficient determined in the range from 0 to1.

The value of the coefficient *p*_*r*_[18] is determined once for the Equation (2) and is 0.9 (it was determined arbitrarily). Depending on its selection (*p*_*r*_), the number of pixels that influence the calculation of the median is changed. An increase in the value of *p*_*r*_ increases the number of pixels from which the median is calculated. Equations (2) and (3) by selecting *p*_*r*_ enable correct calculations of the RPE layer individual points only if there is one cluster. In other cases, when there are two or more clusters, the calculations are more difficult. These are cases where the RPE layer is not the brightest layer for the analysed column. Such situations are very rare. The specificity of the Equations (2) and (3) enables to receive values equal to *M* (last row) for cases where none of the pixels is larger than *max*_*m* ∈ (1,*M*)_*L*_*MED*_(*m*, *n*) * *p*_*r*_ for the analysed column.

Based on the course of *y*_*RPE*_ '' (*n*) (hereinafter abbreviated to *y*_*RPE*_), the image *L*_*RPE*_ was created in the following way:

(4) $$L_{\mathrm{RPE}}\left(m,n\right)=\left\{\begin{array}{c} L_{\mathrm{MED}}\left(m,n\right)\;\mathrm{if}\;m\geq y_{\mathrm{RPE}}\left(n\right)\\ \begin{array}{cc} \begin{array}{cc} \begin{array}{cc} \begin{array}{ccc} -1 & & \mathrm{others} \end{array} & \end{array} & \end{array} & \end{array} \end{array}\right)$$

The values of “-1” added to the matrix *L*_*RPE*_ enable to distinguish the area above the RPE layer from the pixels beneath the RPE with a value of “0”. The final element of image pre-processing is affine transformation of the image *L*_*RPE*_ to the image *L*_*CHO*_ containing only the interesting area of the choroid Figure 2. The image *L*_*CHO*_ includes all the pixels of the image *L*_*RPE*_ from the layer *y*_*RPE*_(*n*) to the last row (*M*). The remaining space in the matrix *L*_*RPE*_ is filled with the values “-1”. Thus prepared image *L*_*CHO*_ with a resolution *M*_*C*_×*N*_*C*_ is subjected to appropriate analysis and processing as described in the next sub-section.

### Image processing

The input image *L*_*CHO*_ shown in Figure 2 provides the basis for further processing. The specific properties of optical scanners as well as the object (eye) specificity contribute to the fact that brightness uniformity correction is necessary for further processing. For this purpose, the image *L*_*MEAN*_, which results from filtration with an averaging filter with a mask *h*_*2*_ sized *M*_*h2*_×*N*_*h2*_ = 30×30 pixels, was subtracted from the image *L*_*CHO*_. As a result, the image *L*_*CHOM*_ was obtained, i.e.:

(5) $$\begin{array}{l} L_{\mathrm{CHOM}}\left(m,n\right)=L_{\mathrm{CHO}}\left(m,n\right)\\ \;-\frac{1}{M_{h2}\cdot N_{h2}}\sum_{m_{2}=1}^{M_{h2}}\sum_{n_{2}=1}^{N_{h2}}\left(L_{\mathrm{CHO}}\left(m+m_{2}-\frac{M_{h2}}{2},n+n_{2}-\frac{N_{h2}}{2}\right)\cdot h_{2}\left(m_{2},n_{2}\right)\right)\\ \; \end{array}$$

for *m*∈(*M*_*h2*_/2, *M*_*C*_-*M*_*h2*_/2) and *n*∈(*N*_*h2*_/2, *N*_*C*_-*N*_*h2*_/2).

The size of the mask *h*_*2*_ is closely related to the size of objects subjected to detection. In the case of the diseases listed in Table 1, the maximum size of objects is 20×20 pixels. Therefore, it was assumed that the size of the mask *h*_*2*_ must be at least twice as large. This is necessary to carry out the removal of uneven brightness. Further processing steps are related to the detection of objects with the use of morphological operations. For these operations, it is necessary to define the binary image *L*_*BW*_. This image contains information on the location of pixels of the image *L*_*CHOM*_ derived from the image *L*_*RPE*_ i.e.:

(6) $$L_{\mathrm{BW}}\left(m,n\right)=\left\{\begin{array}{c} \begin{array}{cccc} 1 & \;\mathrm{if} & L_{\mathrm{RPE}}\left(m,n\right)=-1 & \end{array}\\ \begin{array}{llll} \begin{array}{llll} 0 & \mathrm{others} & & \end{array} & & & \end{array} \end{array}\right)$$

The proper steps of image processing are related to sequential morphological analysis. Morphological opening operations were performed on the image *L*_*CHOM*_. The size of the structural element *SE*_*i*_ was varied every 2 pixels in the range from 3×3 to 11×11 pixels (where the subscript *i* indicates the size, i.e.: *SE*_*3*_ is a structural element sized 3×3 etc.). An opening operation was carried out for every *i*-th size of the symmetric structural element *SE*, i.e.:

(7) $$L_{Oi}=\min_{SE_{i}}\left(\max_{{}_{SE_{i}}}\left(L_{\mathrm{CHOM}}\right)\right)\cdot L_{\mathrm{BW}}$$

These images (*L*_*Oi*_) after normalization to the range 0 to 1 may be subjected to binarization at a constant threshold *p*_*r*_.

(8) $$L_{\mathrm{Bi}}=\left\{\begin{array}{ccc} 1 & \mathrm{if} & \left(L_{\mathrm{Oi}}-\min_{L_{\mathrm{Oi}}}\left(L_{\mathrm{Oi}}\right)\right)\;/\max_{L_{\mathrm{Oi}}}\left(L_{\mathrm{Oi}}-\min_{L_{\mathrm{Oi}}}\left(L_{\mathrm{Oi}}\right)\right)\\ 0 & \mathrm{others} & \end{array}<\mathrm{Pr}\right)$$

The value of the threshold *p*_*r*_ is fixed at 0.5 due to the previously performed operations of removing uneven brightness and standardization. The images *L*_*Bi*_ require adjustments related to the removal of small artefacts and holes in objects. This process was carried out using relationships of conditional erosion and dilation of the binary image *L*_*Bi*_. In the case of a symmetrical structural element *SE*_*i*_, relationships of conditional erosion and dilation [19] are simplified to the following form:

(9) $$\begin{array}{l} L_{E\left(C\right)}\left(m,n\right)=\\ =\left\{\begin{array}{lll} L_{\mathrm{Bi}}\left(m,n\right) & \mathrm{for} & \left(1-p_{\mathrm{we}}\right)\cdot p_{\mathrm{mn}}\left(m,n\right)\leq s_{\mathrm{re}}\left(m,n\right)\\ \min_{m_{\mathrm{SEi}},n_{\mathrm{SEi}}\in \mathrm{SEi}}\left(L_{\mathrm{Bi}}\left(m+m_{\mathrm{SEi}},n+n_{\mathrm{SEi}}\right)\right) & \mathrm{for} & \left(1-p_{\mathrm{we}}\right)\cdot p_{\mathrm{mn}}\left(m,n\right)>s_{\mathrm{re}}\left(m,n\right) \end{array}\right) \end{array}$$

(10) $$\begin{array}{l} L_{D\left(C\right)}\left(m,n\right)=\\ =\left\{\begin{array}{lll} L_{\mathrm{Bi}}\left(m,n\right) & \mathrm{for} & \left(p_{\mathrm{wd}}+1\right)\cdot p_{\mathrm{mn}}\left(m,n\right)\geq s_{\mathrm{rd}}\left(m,n\right)\\ \max_{m_{\mathrm{SEi}},n_{\mathrm{SEi}}\in \mathrm{SEi}}\left(L_{\mathrm{Bi}}\left(m-m_{\mathrm{SEi}},n-n_{\mathrm{SEi}}\right)\right) & \mathrm{for} & \left(p_{\mathrm{wd}}+1\right)\cdot p_{\mathrm{mn}}\left(m,n\right)<s_{\mathrm{rd}}\left(m,n\right) \end{array}\right) \end{array}$$

where:

*L*_*E*(*C*)_(*m,n*) – the resulting binary image after subjecting the image *L*_*Bi*_ to conditional erosion,

*L*_*D*(*C*)_(*m,n*) – the resulting binary image after subjecting the image *L*_*Bi*_ to conditional dilation,

*p*_*we*_ – constant erosion effectiveness,

*p*_*wd*_ – constant dilation effectiveness,

*p*_*mn*_(*m,n*) – threshold dependent on the coordinates *m, n*,

*s*_*re*_ – the mean value of the analysed area for erosion,

*s*_*rd*_ – the mean value of the analysed area for dilation.

The mean values *s*_*re*_, *s*_*rd*_ for erosion and dilation respectively were calculated from the following equations:

(11) $$s_{\mathrm{re}}\left(m,n\right)=\sum_{m_{\mathrm{SEi}}=1}^{M_{\mathrm{SEi}}}\sum_{n_{\mathrm{SEi}}=1}^{N_{\mathrm{SEi}}}\frac{L_{\mathrm{Oi}}\left(m+m_{\mathrm{SEi}},n+n_{\mathrm{SEi}}\right)}{M_{\mathrm{SEi}}\cdot N_{\mathrm{SEi}}}\;$$

(12) $$s_{\mathrm{rd}}\left(m,n\right)=\sum_{m_{\mathrm{SEi}}=1}^{M_{\mathrm{SEi}}}\sum_{n_{\mathrm{SEi}}=1}^{N_{\mathrm{SEi}}}\frac{L_{\mathrm{Oi}}\left(m-m_{\mathrm{SEi}},n-n_{\mathrm{SEi}}\right)}{M_{\mathrm{SEi}}\cdot N_{\mathrm{SEi}}}\;$$

The constants *p*_*we*_ and *p*_*wd*_ determining the effectiveness of erosion and dilation respectively take the following values:

(13) $$\begin{array}{l} p_{\mathrm{we}}\in \left\{-1.0,-0.9,\ldots ,-0.1,0.0,0.1,\ldots ,0.9,1.0\right\},\\ p_{\mathrm{wd}}\in \left\{-1.0,-0.9,\ldots ,-0.1,0.0,0.1,\ldots ,0.9,1.0\right\} \end{array}$$

The choice of this range results from the condition of the left side of inequality, i.e.:

(14) $$\underset{I}{\underset{︸}{\left(1-p_{\mathrm{we}}\right)\cdot p_{\mathrm{mn}}\left(m,n\right)}}>\underset{\mathrm{II}}{\underset{︸}{s_{\mathrm{re}}\left(m,n\right)}}$$

**Figure 3:**
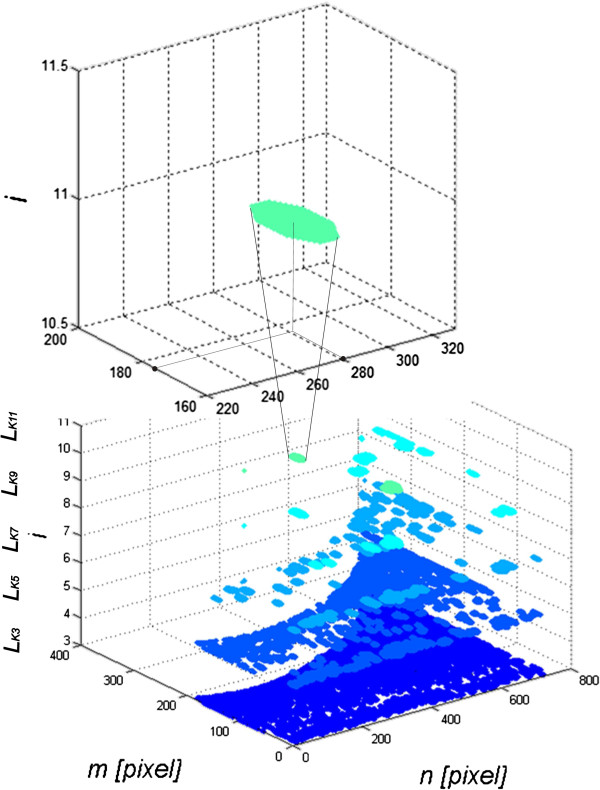
**Image analysis of the sequence of images *L***_***Ki***_**.** Subsequent results are shown for *i*∈(3, 5, 7, 9, 11). For each image *L*_*Ki*_ and thus for each *i* the values of the features from *w*(1) to *w*(20) are calculated. For example, one of the objects whose coordinates of the centre of gravity are calculated is shown on the top of the zoom – in this case, they are (176, 281). The features *w*(6) to *w*(16) are the mean value of gravity centre coordinates of all objects in the image *L*_*Ki*_.

**Figure 4:**
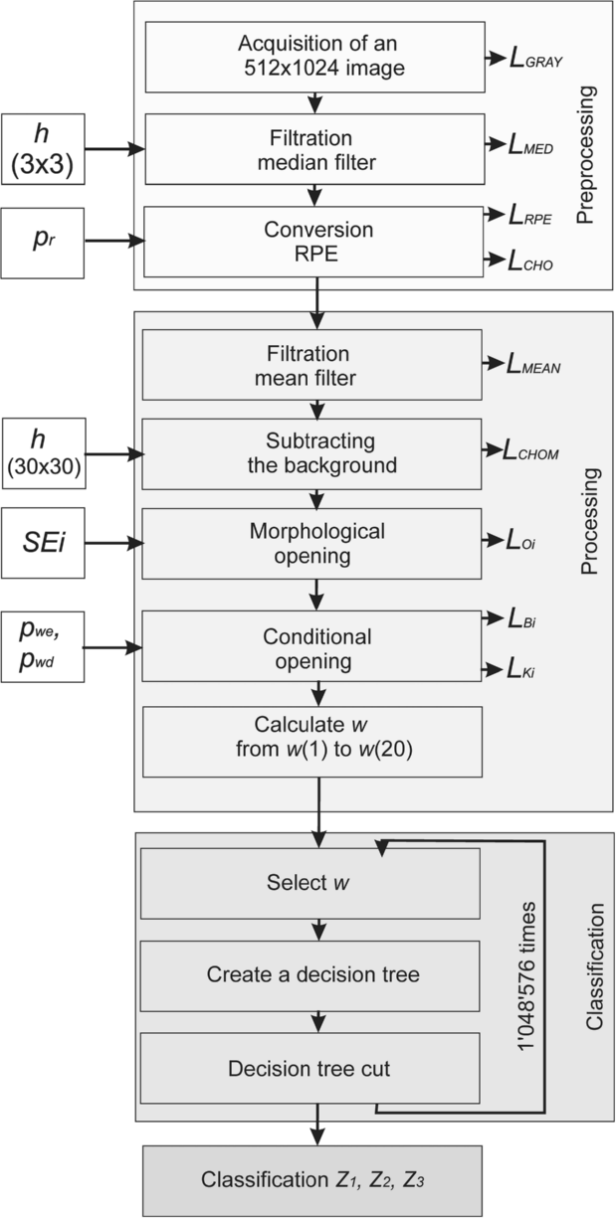
**The block diagram of the algorithm.** The block diagram is divided into three main parts, namely image pre-processing using the known techniques of image analysis and processing, the principal analysis of images proposed by the authors and the classifier based on decision trees. The individual processing blocks are described in detail in the paper.

The values of *p*_*mn*_ are in the range from 0 to 1, whereas the values of (1–*p*_*we*_) should be non-negative in the range from 0 to 2. The values of (1–*p*_*we*_) and (1 + *p*_*wd*_) for *p*_*we*_ *= p*_*wd*_ = 0 are equal to 1, which means high intensity of conditional operations. For the other values of the thresholds *p*_*we*_ and *p*_*wd*_, for example for *p*_*we*_ *= p*_*wd*_ = 1, there is a complete lack of effectiveness of erosion operations and significant effectiveness of dilation (the impact of selection of *p*_*we*_ and *p*_*wd*_ values is presented later in this section). However, very often *p*_*mn*_(*m,n*) *=* const, irrespective of the location (*p*_*mn*_≠*f*(*m,n*)). Adopting *p*_*mn*_(*m,n*) = const is due to the nature of conditional operations, where in a general case a condition may not only be dependent on the mean values of *s*_*re*_ and *s*_*rd*_, but also on other values of the pixel saturation degree. These special properties of conditional dilation and erosion enable to obtain effective correction of the quality of the input images *L*_*Bi*_. Sequential execution of conditional dilation and erosion (in this case, three times) allows to obtain corrected images *L*_*Ki*_ (Figure 3). The shape of the structural element *SEi* adopted in all of these relationships was as a circle of a pre-specified size because of the shape of the recognized objects. For each image *L*_*Ki*_, characteristics were determined for each object. These features include:

*w*(1) to *w*(5) - number of objects in the image *L*_*Ki*_ for *i*∈(3, 5, 7, 9, 11),

*w*(6) to *w*(10) - the average position of the centre of gravity in the *x*-axis for all the objects in the image *L*_*Ki*_ for *i*∈(3, 5, 7, 3, 11),

*w*(11) to *w*(15) - the average position of the centre of gravity in the *y*-axis for all the objects in the image *L*_*Ki*_ for *i*∈(3, 5, 7, 9, 11),

*w*(16) to *w*(20) - standard deviation of the mean brightness of pixels of all the objects in the image *L*_*Ki*_ for *i*∈(3, 5, 7, 9, 11),

These features were selected taking into account medical conditions. They are related to the location of vascular lesions from *w*(6) to *w*(15), uniformity of brightness distribution within these changes from *w*(16) to *w*(20) and the area of changes for the appropriate number of objects from *w*(1) to *w*(5). These characteristics form the basis for building a classifier using decision trees (Figure 4 shows the block diagram of the algorithm).

## Results

The values of the 20 features obtained (four different types) are further used to build a decision tree. An ophthalmologist divided more than 1′000 images into three groups (*Z*_*1*_*, Z*_*2*_ or *Z*_*3*_ - Table 1). The division into groups *Z*_*1*_*, Z*_*2*_ or *Z*_*3*_ was performed manually by an ophthalmologist. As a result, the following frequency of occurrence of choroidal imaging was obtained: the group *Z*_*1*_ included 23% of all cases, the group *Z*_*2*_ included 44% of all cases, and the group *Z*_*3*_ included 33% of all cases. This corresponds to 233, 436 and 331 images respectively. Whereas the division into learning, validation and test groups had the following proportions: 60%, 20% and 20% respectively. These groups were formed after rejecting the images with a mixed character of the changes observed - the type of the disease was not a criterion for exclusion here. A variety of overlapping diseases can be found in the excluded images. The cases of spatially invisible layer of choroid, images resulting from the errors in the acquisition or lacking a visible layer of RPE (for various reasons) were also excluded. Due to the fact that these are retrospective studies, the excluded images did not often cover the full range of the choroid, were deliberately obscured or distorted at the acquisition stage.

**Figure 5:**
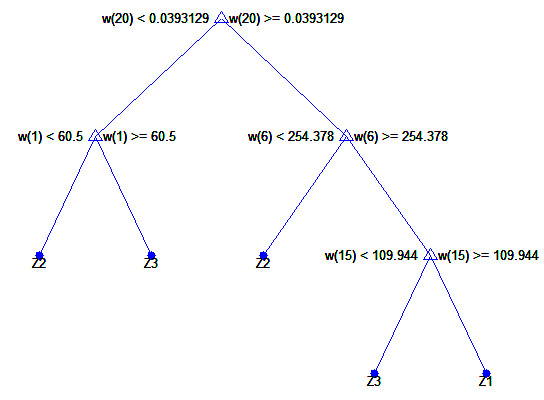
**The complete classification tree.** The decision tree was created for the classification of imaging types *Z*_*1*_*, Z*_*2*_*, Z*_*3*_ for the features *w*(1) to *w*(20). The characteristic elements such as the presence of the feature *w*(20) in the first node of the tree and successively features *w*(1), *w*(6) and *w*(15) for the other nodes are visible here. The results obtained on this basis are, for example, *SPC*_*2*_ = 0.56, *TPR*_*2*_ = 0.86, *ACC*_*2*_ = 0.70 for *Z*_*2*_.

**Figure 6:**
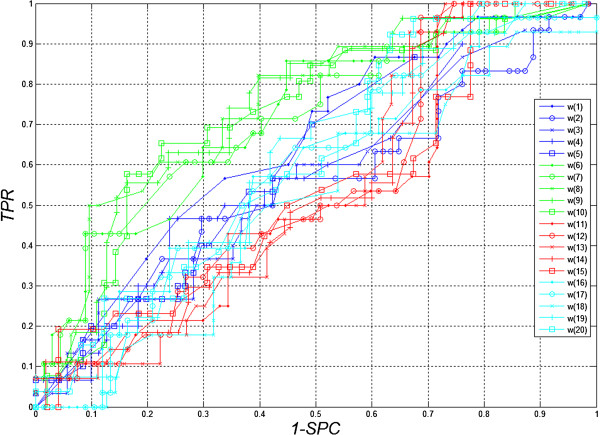
**ROC Chart (Receiver Operating Characteristic).** The graphs show the impact of threshold values for individual features *w*(1) to *w*(20) for the different imaging types *Z*_*1*_*, Z*_*2*_*, Z*_*3*_. A negligible impact of the features *w*(11) and *w*(20) on the obtained results can be inferred here. In addition, sensitivity of features to changes in decision-making threshold values is the smallest for the feature *w*(11) and the highest for *w*(8).

**Figure 7:**
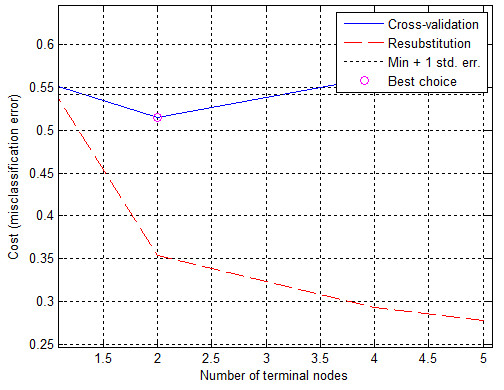
**The graph of the cost relationships (misclassification error) as a function of stratified cross-validation and resubstitution.** The blue shows the cross-validation error. The best choice, the place of decision tree trimming is circled as the closest cut below the minimum error value plus a mean square error (min + 1 std. err).

The nodes of the decision tree are different features from *w*(1) to *w*(20), the branches are the values corresponding to these attributes, and the leaves make individual decisions – type identification (*Z*_*1*_*, Z*_*2*_ or *Z*_*3*_ – Table 1). In all cases, a non-parametrical algorithm creating CART (Classification and Regression Trees) binary trees was used as the method for decision tree induction. An increase in the nodes purity was used as the criterion assessing the quality of CART divisions. The Gini index was used as the measure of nodes impurity. The tree creation was not limited by a minimum number of vectors in a node. As the considerations apply to the construction of a classifier based on the knowledge base, *w*(1) to *w*(20) features, a preliminary prepared tree Figure 5 was built based on the full information, using the training group. The results of the classification for the complete decision tree for the test group as follows:

–for *Z*_*1*_ – *SPC*_*1*_ = 0.74, *TPR*_*1*_ = 0.32, *ACC*_*1*_ = 0.64,

–for *Z*_*2*_ – *SPC*_*2*_ = 0.56, *TPR*_*2*_ = 0.86, *ACC*_*2*_ = 0.70,

–for *Z*_*3*_ – *SPC*_*3*_ = 0.88, *TPR*_*3*_ = 0.097, *ACC*_*3*_ = 0.63.

(where: *SPC* = *TN*/(*FP* + *TN*) – specificity, *TPR* = *TP*/(*TP* + *FN*) – sensitivity, *TN* – true negative, *TP* – true positive, *FN* – false negative, *FN* – false positive, subscripts “1”, “2” or “3” indicate the type of images). A closer analysis of the resulting decision tree enables, at this stage, rough assessment of the importance of individual features. For this form of the complete decision tree, there occur only features *w*(1), *w*(6), *w*(15) and *w*(20). Additional information is provided by the ROC graph (Receiver Operating Characteristic) designated for *Z*_*1*_, *Z*_*2*_, *Z*_*3*_ to assess the impact of individual features from *w*(1) to *w*(20) separately. This graph (ROC) is shown in Figure 6. Based on the ROC graph and the complete decision tree, it can be concluded that there is a negligible impact of the features *w*(11) to *w*(20) on the obtained results. Additionally, the sensitivity of features to changes in the decision-making threshold value is the smallest for the feature *w*(11), and the highest for *w*(8) (Figure 6). For this reason, decision trees were created for each configuration of features, thereby forming 2^20^ full decision trees (unpruned trees) and the same number of cut decision trees (over two million decision trees in total - 2′097′152). Figure 7 shows the graph of cost relationships (misclassification error) as a function of stratified cross-validation and resubstitution. The graph was also shown by computing a cutoff value that is equal to the minimum cost plus one standard error. The best level computed by the classregtree test method is the smallest tree under this cutoff (best level = 0 corresponds to the unpruned tree). The best results obtained for the complete decision tree are shown in Table 2. The best results of *ACC*_*2*_ *=* 0.83 were obtained for the features *w*(1), *w*(2), *w*(3) and *w*(4).

**Table 2:** The three top results obtained for different configurations of the features *w*(1) to *w*(20)* for the complete decision tree

	***Z***_***1***_			***Z***_***2***_			***Z***_***3***_		
***w*/N°**	**1**	**2**	**3**	**1**	**2**	**3**	**1**	**2**	**3**
***w*(1)**	0	0	0	1	1	0	1	1	1
***w*(2)**	0	0	0	1	1	0	0	0	1
***w*(3)**	0	0	0	1	1	0	1	0	0
***w*(4)**	1	1	1	1	1	0	0	0	0
***w*(5)**	0	0	0	0	1	1	0	0	0
***w*(6)**	1	1	1	0	0	0	1	0	0
***w*(7)**	0	0	0	0	0	1	0	1	1
***w*(8)**	0	1	0	0	0	1	0	0	0
***w*(9)**	0	0	0	0	0	0	0	0	0
***w*(10)**	0	0	0	0	0	0	0	0	0
***w*(11)**	0	0	0	0	0	0	0	0	0
***w*(12)**	0	0	0	0	0	0	0	0	0
***w*(13)**	0	0	1	0	0	0	0	0	0
***w*(14)**	0	0	0	0	0	0	0	0	0
***w*(15)**	1	1	1	0	0	0	0	0	0
***w*(16)**	0	0	0	0	0	1	0	0	0
***w*(17)**	0	0	0	0	0	1	0	0	0
***w*(18)**	0	0	0	0	0	0	0	0	0
***w*(19)**	0	0	0	0	0	0	0	0	0
***w*(20)**	0	0	0	0	0	0	0	0	0
***ACC***_***i***_	0.73	0.73	0.73	0.83	0.83	0.83	0.69	0.69	0.69

*The occurrence of a given feature *w* is indicated by “1” and its lack by “0”.

**Figure 8:**
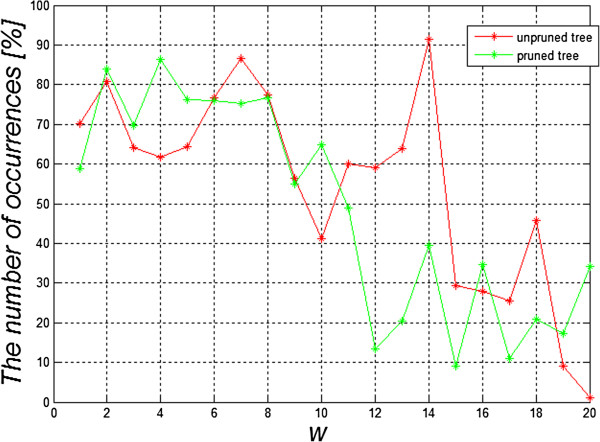
**The graph of occurrence frequency of individual features *w*(1) to *w*(20) for the complete decision tree and the cut one for the first top 1′000 results.** The graph shows that the highest occurrence concerns the feature *w*(14) for the complete decision tree and the feature *w*(4) for the cut decision tree. The occurrence frequency of the features corresponds with the results shown in Tables 2 and 3.

In the classification of groups *Z*_*1*_, *Z*_*2*_, *Z*_*3*_ the features from *w*(9) to *w*(12) as well as from *w*(18) to *w*(20) did not occur. This means that their influence can be neglected for the best classifications (top 3 results). These features (from *w*(9) to *w*(12) as well as from *w*(18) to *w*(20)) define the average location of the centre of gravity for *i* = 9 and 11 in the *x*-axis, for *i* = 3, 5 in the *y*-axis and the standard deviation of the average brightness of pixels of all objects for *i* = 9 and 11. The same results of *ACC*_2_ were obtained for various configurations of the features *w*(1), *w*(2), *w*(3) and *w*(4) or *w*(5), *w*(7), *w*(8), *w*(16) and *w*(17), which is also quite interesting (Table 2). The best results for the cut decision tree are shown in Table 3. *ACC*_*2*_ *=* 0.82, obtained for the features *w*(1), *w*(4) *w*(5), *w*(7) *w*(8), *w*(12) and *w*(15), is the best result for the cut decision tree. The results for the groups *Z*_*1*_, *Z*_*2*_, *Z*_*3*_ confirm that the features from *w*(17) to *w*(20) have the smallest influence. The summary frequency chart of the occurrence of individual features from *w*(1) to *w*(20) for the cut and complete decision trees for the first 1′000 best results is interesting (Figure 8). It shows the greatest frequency of occurrence of the feature *w*(14) for the complete decision tree and the feature *w*(4) for the cut decision tree. From a practical standpoint, however, minimizing the number of features occurring in the classification seems to be the most vital. Therefore, when analysing only the three top results for the cut decision tree, one feature for *Z*_*1*_, six features *Z*_*2*_ and one feature for *Z*_*3*_ are obtained. This means that only the group *Z*_*2*_ requires the largest number of features in the classification. This information is essential for optimizing the computational complexity of the algorithm. The analysis time for a single image does not exceed one second for the Pentium 4 CPU 3.0 GHz, 8GB RAM.

**Table 3:** **The three top results obtained for different configurations of the features *w*(1) to *w*(20) for the cut decision tree**

	***Z***_***1***_			***Z***_***2***_			***Z***_***3***_		
***w*/N°**	**1**	**2**	**3**	**1**	**2**	**3**	**1**	**2**	**3**
***w*(1)**	0	1	0	1	1	1	1	0	0
***w*(2)**	0	0	1	0	0	0	1	0	0
***w*(3)**	0	0	0	0	1	1	0	1	0
***w*(4)**	1	1	1	1	0	0	0	0	1
***w*(5)**	0	0	0	1	0	1	0	0	0
***w*(6)**	0	0	0	0	1	1	0	0	0
***w*(7)**	0	0	0	1	0	0	0	0	0
***w*(8)**	0	0	0	1	0	0	0	0	0
***w*(9)**	0	0	0	0	1	1	0	0	0
***w*(10)**	0	0	0	0	0	0	0	0	0
***w*(11)**	0	0	0	0	0	0	0	0	0
***w*(12)**	0	0	0	1	1	1	0	0	0
***w*(13)**	0	0	0	0	0	0	0	0	0
***w*(14)**	0	0	0	0	0	0	0	0	0
***w*(15)**	0	0	0	1	0	0	0	0	0
***w*(16)**	0	0	0	1	1	1	0	0	0
***w*(17)**	0	0	0	0	0	0	0	0	0
***w*(18)**	0	0	0	0	0	0	0	0	0
***w*(19)**	0	0	0	0	0	0	0	0	0
***w*(20)**	0	0	0	0	0	0	0	0	0
***ACC***_***i***_	0.76	0.76	0.76	0.82	0.81	0.81	0.68	0.68	0.68

### Comparison with other authors’ results

The analysis of the choroidal layer is dealt with in a number of publications [20-23]. In most cases, however, it is qualitative analysis. It includes the analysis of the choroidal layer thickness [20] or analysis with the use of Heidelberg Eye Explorer software [21]. In the first case, central serous chorioretinopathy case-series is presented. The following results were obtained – mean subfoveal choroidal thickness was significantly (significance level *p*_*sl*_ = 0.04) larger in the affected eyes (455 ± 73 μm) than in the contralateral unaffected eyes (387 ± 94 μm), in which it was significantly (*p*_*sl*_ = 0.005) larger than in the control group (289 ± 71 μm). The control group and the group of patients in this case [20] comprised 15 + 15 patients. The largest vessel diameter was significantly (*p*_*sl*_ < 0.001, correlation coefficient: 0.73) correlated with the thickness of the total choroid. A similar small number of patients was analysed in [21]. The described software enables to measure some features of the choroid. However, this method is fully manual. Another interesting analysis is shown by Mrejen S. in [22]. The results obtained there concern the use of various imaging techniques in the diagnosis of the choroid. An automatic method for the analysis of the choroid is shown in the work of Park S.Y. [23]. The analysis presented there concerned only determination of the location of the selected layers in the tomographic image of the fundus. In addition, the correlation between the early treatment diabetic retinopathy study and EDI (enhanced depth imaging) was demonstrated. The automatically measured retinal thickness and volume of 9 early treatment diabetic retinopathy study subfields with conventional and EDI raster scan showed an intraclass correlation coefficient of 0.861 to 0.995 and 0.873 to 0.99 respectively. The number of analyzed cases was 35 patients with chorioretinal diseases and 20 healthy subjects. In this paper, the number of cases is much higher and the image analysis and processing are fully automatic. It seems that useful results can be obtained even with non-EDI SOCT. However, it should be further tested with other devices.

### Summary

This paper shows a new fully automated method for the analysis of choroidal images. A division into three types of choroidal images was proposed. The created decision tree enabled to obtain satisfactory classification results. For example, for the classification of images *Z*_*2*_, the accuracy *ACC*_*2*_ was 0.81 (pruned tree). Additionally, the features which have the greatest impact on the classification efficiency were characterized (creating more than 2 million decision trees). These are the characteristics from *w*(1) to *w*(9), *w*(12), *w*(15) and *w*(16) which are responsible for the number of objects in the scene and the average position of the centre of gravity in the *x*-axis for *i* = 3, 5, 7, 9 and the average position of the centre of gravity in the *y*-axis for *i =* 5, 11 and STD for *i* = 3. In addition, it was shown that for the adopted classifier of the cut decision tree, for the top 1′000 results the feature *w*(4) was most common. It was also noted that the different locations of scanning differentiate the images of the choroid but the classification result remains the same. Foveal location, however, has the greatest ability to differentiate changes. Individual B-scans located on the macula periphery are closer to each other. The proposed algorithm for image analysis and the classifier were implemented in the C language. Currently, the application is further studied in the Clinical Department of Ophthalmology in District Railway Hospital, Medical University of Silesia in Katowice, Poland.

The presented methodology of the procedure does not cover a large range of opportunities offered by modern methods of image analysis and processing. Currently, there is ongoing work on the optimization of the algorithm in terms of computational complexity. Moreover, its aim is to replace some of the time-consuming functions with some other more efficient ones. In particular, the possibility of applying different methods of texture analysis [23-31], Boolean function [32] or other methods of image analysis [33,34] or the impact of other factors [35-41] is considered. Other types of classifiers ensuring the analysis of other characteristics acquired from images are particularly taken into account.

## Consent

Written informed consent was obtained from the patient for the publication of this report and any accompanying images.

## Abbreviations

RPE: Retina pigment ephitelium; CRA: Central retinal artery; FAZ: Foveal avascular zone; ROC: Receiver operating characteristic; ACC: Accuracy; SPC: Specificity; TP: True positive; TN: True negative; FN: False negative; FP: False positive; EDI: Enhanced depth imaging.

## Competing interests

The authors declare that they have no competing interests.

## Authors’ contributions

RK suggested the algorithm for image analysis and processing, implemented it and analysed the images. ST, EW, ZW performed the acquisition of the OCT images and consulted the obtained results. All authors have read and approved the final manuscript.
